# Development of mobile technologies for the prevention of cervical cancer in Santiago, Chile study protocol: a randomized controlled trial

**DOI:** 10.1186/s12885-017-3870-8

**Published:** 2017-12-13

**Authors:** McKenzie C. Momany, Javiera Martinez-Gutierrez, Mauricio Soto, Daniel Capurro, Francis Ciampi, Beti Thompson, Klaus Puschel

**Affiliations:** 1 Department of Family Medicine Pontificia, Universidad Católica de Chile, Santiago, Chile; 2 Department of Internal Medicine Pontificia, Universidad Católica de Chile, Santiago, Chile; 3 Centro de Salud Familiar El Roble, Santiago, Chile; 4 Fred Hutchinson Cancer Research Center, Seattle, Washington USA; 5 School of Medicine, University of Washington, Seattle, Washington USA

**Keywords:** Chilean women, Pap test, Cervical cancer screening, Cancer disparities

## Abstract

**Background:**

In Chile, more than 500 women die every year from cervical cancer, and a majority of Chilean women are not up-to-date with their Papanicolau (Pap) test. Mobile health has great potential in many health areas, particularly in health promotion and prevention. There are no randomized controlled trials in Latin America assessing its use in cervical cancer screening. The ‘Development of Mobile Technologies for the Prevention of Cervical Cancer in Santiago, Chile’ study aims to determine the efficacy of a text-message intervention on Pap test adherence among Chilean women in the metropolitan region of Santiago.

**Methods/design:**

This study is a parallel randomized-controlled trial of 400 Chilean women aged 25–64 who are non-adherent with current recommendations for Pap test screening. Participants will be randomly assigned to (1) a control arm (usual care) or (2) an intervention arm, where text and voice messages containing information and encouragement to undergo screening will be sent to the women. The primary endpoint is completion of a Pap test within 6 months of baseline assessment, as determined by medical record review at community-based clinics. Medical record reviewers will be blinded to randomization arms. The secondary endpoint is an evaluation of the implementation and usability of the text message intervention as a strategy to improve screening adherence.

**Discussion:**

This intervention using mobile technology intends to raise cervical cancer screening adherence and compliance among a Chilean population of low and middle-low socioeconomic status. If successful, this strategy may reduce the incidence of cervical cancer.

**Trial registration:**

Clinicaltrials.gov NCT02376023 Registered 2/17/2015.

First participant enrolled Feb 22nd 2016.

**Electronic supplementary material:**

The online version of this article (10.1186/s12885-017-3870-8) contains supplementary material, which is available to authorized users.

## Background

Cervical cancer is the third leading cause of death of women worldwide. In 2020 more than 315,000 women are estimated to die due to cervical cancer; more than 85% of these deaths will be in developing countries. In 2015, an estimated 88,000 new women were diagnosed with cervical cancer in the Americas, with more than 38,000 deaths estimated that same year [1]. In Chile, more than 500 women die every year from cervical cancer. In Chile, only 59% of the population is up-to-date with their Pap test, and this figure has not changed in the last 10 years [2]. The distribution of cervical cancer, like that of other cancers, follows a pattern of inequality in that women of lowest socioeconomic status are those most affected [3, 4].

Mobile technologies have increased exponentially in the last few years [5]; in 2009, mobile telephones could be found in more than 90% of Chilean homes and were widely distributed across all socioeconomic levels. While inequalities still remain in this area, they are much less profound than with other technologies; its distribution varies between 97% in high SES and 82.8% in low SES [6, 7]. Because of this technological explosion, mobile health (mHealth), or “medical and public health practice supported by mobile devices” [8], has great potential in many health areas such as promotion and prevention [9]. Overall interest in mobile health is widespread. The World Health Organization’s (WHO) report on mHealth in 2011 [8] states that mobile health strategies exist in at least 75% of the countries that belong to the WHO in each region. According to the European Commission in its program “Digital Agenda for Europe”, mHealth has the potential to reduce inequalities regarding the delivery of health services, to empower patients to control their own health, and to improve the cost-effectiveness of health care delivery [10].

There is a lack of evidence supporting mHealth in cancer prevention. Some evidence exists that shows that mobile health is an effective strategy for treatment adherence for prenatal care and those with HIV and tuberculosis [11–14]. In cancer prevention, a study in Botswana described how community workers were trained to use mobile cameras to send images of possible cervical cancer to expert gynecologists located remotely. The study concludes mHealth could be a powerful tool for cervical cancer screening [15]. Nevertheless, there are no reported randomized controlled trails in Latin America of using mobile health technologies in cancer prevention.

The aims of this study are: 1. To ascertain the efficacy of an intervention using mobile technologies on Pap test adherence compared to a control condition; and 2. To evaluate the implementation and usability of this intervention in three health centers of the South East Metropolitan Health District of Santiago, Chile.

## Methods/design

The ‘Development of Mobile Technologies for the Prevention of Cervical Cancer in Santiago, Chile’ or “Messages for your health” is a parallel randomized controlled trial with both a qualitative and quantitative phase. In the qualitative phase, we aimed to acquire information to design a suitable intervention using mobile technologies for our study population. Six focus groups (two in each health center) were carried out with women who received care at eligible health care centers in order to determine the barriers and facilitators to implementing a text message intervention. An additional three focus groups were carried out with midwives at the health care centers in order to characterize the usual care that women receive. (In Chile, midwives are in charge of women’s primary care). This phase was completed in March 2015 and served as basis for the developing the quantitative phase of the actual mHealth intervention. Focus group guides in English and Spanish can be reviewed as Additional file 1.

**Fig. 1:**
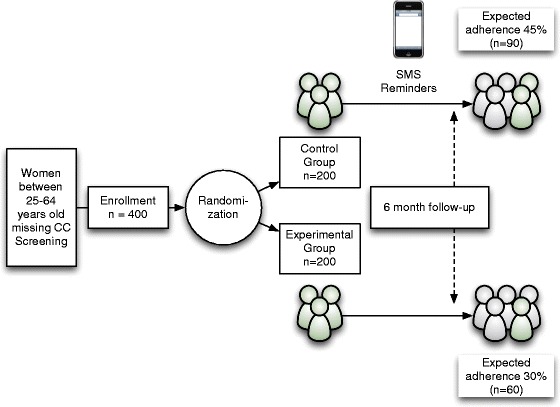
Study protocol. Participants´ randomization scheme

In the quantitative phase, participants will be randomized into a control/usual care arm or a text message intervention arm (Fig. 1). The messages will educate participants about the importance of cervical cancer screening and encourage them to receive a Pap test. The study is being conducted in La Pintana, Santiago, Chile and has been approved by the Institutional Review Board (IRB) of the Pontificia Universidad Católica de Chile. ID: CEC MED UC 14–213.

### Setting

Chile has a universal health coverage system and local community registries in primary health clinics. The study is being conducted in two health care centers located in the district of La Pintana. The municipality of La Pintana is one of 52 municipalities in the metropolitan region of Santiago. Approximately 30% of the population in La Pintana lives below the poverty level, which is a significantly higher rate than the 14.4% of people living in Chile below the poverty level. The average household income in La Pintana is 635 USD. In Chile it is 1100 USD [16].

Originally the study was to be implemented in three health clinics, two health clinics located in the municipality of La Pintana and one health clinic located in the municipality of Puente Alto.

Both La Pintana clinics serve populations with similar demographics and are funded by the state through a capitated model. The mode of administration differs in that the municipality manages one clinic and the other is managed by a private University.

We chose these clinics since there may be differences in adherence given these two different administrators. The Puente Alto clinic was finally excluded because demographics did not align with La Pintana clinics and efforts to recruit participants were not successful.

With the assistance of the clinics’ health care teams, investigators will identify women non-adherent with Pap testing and review medical records at the end of the study to monitor Pap test screening adherence.

### Participants

The study aims to recruit 400 women who will be enrolled at one of the two participating health care centers. Participating women will be non-adherent with current Chilean recommendations for Pap test screening (they will not have a Pap test within the past 3 years). Eligible women will be between 25 and 64 years of age with no prior history of cervical cancer. Participants must own and use a mobile phone, be free from any mental or physical disabilities that inhibit them from understanding the implications of the study or being able to reach the health clinic for an exam, and cannot be considering relocating within the next year.

### Recruitment of participants

Computerized patient records will be used to obtain basic patient information of women who receive care at the two participating health care centers.

All non-adherent, eligible women registered in the clinic will be appropriate to participate in this study. Those agreeing to participate will be included until we have reached the estimated sample size of 400 participants. Women will be reached by a community health worker at their house or by phone.

Those interested in participating will be told they will be randomized to an intervention arm or a control arm in the project. The community health worker will ask the women to sign an informed consent to participate in the study. Once consent is obtained, the participant will complete a baseline survey. The baseline questionnaire includes items on cervical cancer screening knowledge and attitudes, sociodemographic variables, and cellphone use. Women will receive an estimated 4USD charge in their cell phones as incentive to keep their phone number for as long as the intervention lasts.

### Randomization

Following baseline assessment, women will be randomized to either the intervention or control arm via a computerized program. Investigators and statisticians will be blinded to the allocation groups.

#### Control arm

Participants randomized to the control arm (usual care) will not receive any educational or motivational messages or intervention materials from study staff. Usual care consists of any information on Pap tests and cervical cancer risk reduction typically provided by midwives to all women at the clinics. Women can schedule an appointment for their Pap test in person at the clinics. All services at the clinics, including Pap testing, are free. Usual care may vary slightly across the different participating clinics.

#### mHealth intervention

We chose Nexmo ® as the platform to deliver the mHealth messages given its reliability and low cost. Participants will be sent messages containing information and encouragement to undergo cervical cancer screening. Information will also be provided about health clinic hours and locations. Information and motivational text messages will be delivered twice a week for four months followed by two months of voice messages also twice a week, with the same information. As noted above, specific content, frequency, and message modality (text vs voice message) was determined according to the results of the focus groups and participants’ preferences.

### Primary outcome

The primary outcome is the completion of a Pap test within 6 months of baseline assessment. Participants will be tracked via medical record review as well as through the national database for Pap registry. The difference in Pap testing rate between control and intervention arms will be determined.

### Secondary outcomes

We will assess implementation and usability of a text message intervention by estimating the number of messages sent and received, the answering rate to the voice calls and the stability and reliability of the platform chosen.

### Sample size

We will estimate adherence probabilities to be approximately 0.30 for the usual care arm and 0.45 for the text message intervention arm. Power calculations are based on a sample size of 176 in each of the two study arms, and assume a 10% attrition. A Chi square test will be carried out to compare compliance between the control arm and the intervention arm. The significance level is aimed at 0.05 with a power of 80%.

### Statistical analysis

Pap test adherence will be coded as a binary variable. The intervention will be evaluated based only on Pap test completion at six months after the baseline survey date. Chi square tests of 2 X 2 tables (compliance Yes/No by arm Control vs Text Message Intervention) will be used to determine if the intervention affects the probability of Pap test compliance at follow-up. An expected 400 women (200 per arm to each of 2 arms; control, text message intervention) will be randomized into the study, which will accommodate a 10% loss to follow-up.

We will adjust for age, socioeconomic class, number of children and other relevant demographic and clinical variables.

## Discussion

Cervical cancer screening is a national priority in Chile. Even with free screening nationwide and a cervical cancer screening registry, more than 40% of eligible women do not adhere to cancer screening guidelines. Mobile technologies have permeated widely in Latin America and in Chile but there are no randomized control trials described using these technologies for cancer screening locally.

The ‘Development of Mobile Technologies for the Prevention of Cervical Cancer in Santiago, Chile’ study has the potential to evaluate mobile health as a means to reduce disparities in the incidence of cervical cancer through promoting Pap test adherence to increase early detection. The results of this study will hopefully increase Pap adherence of low SES Chilean women. Determining the effect of an mHealth intervention on screening adherence among low SES Chilean women who are non-adherent with current recommendations for Pap test screening may help with increasing rates of screening adherence nationally. If effective, an mHealth intervention strategy may serve as a means of reducing cervical cancer morbidity and mortality, and could possible be applied to the prevention of other diseases.

## Additional file

Additional file 1: Focus group guides in English and Spanish. (ZIP 226 kb)

## Abbreviations

CESFAM: Centros de Salud Familiar (Family Health Centers)
HIV: Human Immunodeficiency Virus
IRB: Institutional Review Board
MHealth: Mobile health
Pap: Papanicolau
SES: Socioeconomic Status
TB: Tuberculosis
WHO: World Health Organization

## References

[1] Globocan 2012, cancer Incidence, Mortality and Prevalence Worldwie. Retrieved on December 1 from http://globocan.iarc.fr/Pages/burden_sel.aspx.

[2] Defunciones y mortalidad por causas. (n.d.). Retrieved June 4, 2014, from http://www.deis.cl/?p=2541.

[3] Fica A. Prevención del cáncer cérvico-uterino en Chile: Mucha vacuna y poco Papanicolau. Revista Chilena De Infectología. 2014;31(2):196-203. Retrieved June 4, 2014, from http://www.scielo.cl/scielo.php?script=sci_arttext&pid=S0716-10182014000200010&lng=es&tlng=es. doi:10.4067/S0716-10182014000200010. 10.4067/S0716-10182014000200010 24878908

[4] Martínez-Bejarano, R & Martínez-Salgado, C. La mortalidad por cáncer cérvico uterino y de mamá en Colombia y Mexico como expresión de las desigualdades socio-económicas y de genero. III Congreso de la Asociación Latinoamericana de Población, ALAP, Córdoba –Argentina, from 24–26 of September 2008. Retrieved June 4, 2014, from http://www.alapop.org/alap/images/DOCSFINAIS_PDF/ALAP_2008_FINAL_155.pdf.

[5] The World in 2014. ICT facts and figures. ITU world telecommunications. Retrieved June 4, 2014, from http://www.itu.int/en/ITU-D/Statistics/Pages/facts/default.aspx.

[6] Encuesta Nacional de Consumidores de Servicios de Telecomunicaciones. Subsecretaria de Telecomunicaciones 2014. http://www.subtel.gob.cl/wp-content/uploads/2015/04/Presentacion_Final_Sexta_Encuesta_vers_16102015.pdf.

[7] Abonados Móviles. Mobile Subscribers. 2015. Retrieved June 4, 2014, from http://www.subtel.gob.cl/estudios-y-estadisticas/telefonia/.

[8] MHealth: New Horizons for Health through Mobile Technologies. 2011. Global Observatory for EHealth Series, 3. Retrieved June 4, 2014, from http://www.who.int/goe/publications/goe_mhealth_web.pdf.

[9] Aylward D, Leão B, Curioso W, Cruz F (2010). Can you heal me now? Potential (and pitfalls) of mHealth. Americas Quarterly.

[10] Green paper on mobile health (mHealth). European Commission; 2010. Retrieved June 4, 2014, from http://ec.europa.eu/digital-agenda/en/news/green-paper-mobile-health-mhealth.

[11] Vodopivec-Jamsek V, de Jongh T, Gurol-Urganci I, Atun R, Car J. Mobile phone messaging for preventive health care. Cochrane Database Syst Rev. 2012;(12):CD007457. doi:10.1002/14651858.CD007457.pub2. 10.1002/14651858.CD007457.pub2 PMC6486007 23235643

[12] Curioso W, Kepka D, Cabello R, Segura P, Kurth A. Understanding the facilitators and barriers of antiretroviral adherence in Peru: a qualitative study. BMC Public Health. 2010;10:13. doi:10.1186/1471-2458-10-13. 10.1186/1471-2458-10-13 PMC2820472 20070889

[13] Curioso W, Gozzer E, Valderrama M, Rodriguez-Abad J, Villena J, Villena A. Understanding the potential role of cell phones and the Internet to support care for diabetic patients and caregivers in Peru. AMIA 2009 Symposium Proceedings Page, 805. Retrieved June 4, 2014, from http://faculty.washington.edu/wcurioso/Curioso_Understanding_AMIA_2009.pdf.

[14] Curioso W, Gozzer E, Valderrama M, Rodriguez-Abad J, Villena J, Villena A (2009). Uso y percepciones hacia las tecnologías de información y comunicación en pacientes con diabetes en un hospital público del Perú. [Use and perceptions towards information and communication technologies in patients with diabetes in a Peruvian public hospital]. Rev Per Med Exp Sal Pub.

[15] Quinley KE, Gormley RH, Ratcliffe SJ, Shih T, Szep Z, Steiner A (2011). Use of mobile telemedicine for cervical cancer screening. J Telemed Telecare.

[16] OBSERVATORIO SOCIAL. Ministerio de Desarrollo Social. Reporte Comunal: La Pintana, Región Metropolitana. Serie Informes Comunales, N°1. Feb 2014. Retrieved July 2016 from http://observatorio.ministeriodesarrollosocial.gob.cl/indicadores/reportes_com1_2.php.

