# Supplementary material for: Development of mobile technologies for the prevention of cervical cancer in Santiago, Chile study protocol: a randomized controlled trial
Source: BMC Cancer. 2017 Dec 13;17:847. doi: 10.1186/s12885-017-3870-8 (PMC5729241; doi:10.1186/s12885-017-3870-8)
Supplement: Additional file 1: — Focus group guides in English and Spanish. (ZIP 226 kb) [file 12885_2017_3870_MOESM1_ESM.zip › Supplemental Material 1. Focus group guides - EnglishR2.docx]

“MESSAGES FOR YOUR HEALTH” STUDY

FOCUS GROUP GUIDE FOR HEALTH CARE PROFESSIONALS

**Focus Group Objective:**

Understand the barriers and facilitators of a text message intervention to improve Pap adherence rates.

Understand routine care.

**INTRODUCTION (5 MIN)**

**Facilitator steps to follow:**

1. Welcome the group
2. Present the research team
3. Explain the focus group objective, why the focus group participants were invited, and why the study is important.

“**The goal of this focus group is to better understand what you all routinely do to improve Pap adherence, and to hear your opinions regarding what type of messages we should send to your patients. Because of this, we ask that you answer our questions in a sincere manner”.**

1. Explain how the focus group will proceed, including the various themes that will be discussed, how long the focus group will last, and that it will be **recorded**. Explain the focus group rules:
   1. Everybody can and should participate.
   2. Everybody should listen to and respect others’ opinions.
   3. Nobody can “win” or “lose”.
   4. There are no “good” or “bad” answers.
2. Clarify the confidentiality of the focus group, including informed consent, focus group recording, and the use of pseudonyms.

**Participant introduction –** each participant introduces themselves (10 min)

**QUESTIONS (30 MIN)**

**Introduction: We would like to begin by asking questions about the routine care that women who need a Pap receive.**

1. What kinds of promotional activities do you all do to incentivize patients to get a Pap?
2. After you began working as a midwife, did you receive extra training to do Pap smears?
3. What should a text message intervention to improve Pap adherence rates look like? (What should the messages say, how frequently should they be sent, should patients be able to answer them, etc.)
4. What are potential barriers and how could we overcome them?
5. What could be beneficial about this kind of intervention?
6. Is there anything else you would like to add?

**CONCLUSION (3 MIN)**

**Your answers will be very helpful for our study. Before we end, is there anything else you think could help us improve our study?**

**Thank you so much for your honest answers and time!**

“MESSAGES FOR YOUR HEALTH” STUDY

FOCUS GROUP GUIDE FOR FEMALE PATIENTS

**Focus Group Objective:**

Understand the perceptions of women who are not up to date with their Pap smear regarding the use of mobile phones and text messaging as a means for improving Pap adherence.

**INTRODUCTION (5 MIN)**

**Facilitator steps to follow:**

1. Welcome the group
2. Present the research team
3. Explain the focus group objective, why the focus group participants were invited, and why the study is important.

“**The goal of this focus group is to better understand what opinions you all have regarding the use of mobile phones and text messaging in health care. The opinion of each and every one of you is very important because it will help us understand how your use your phones and how mobile technologies can be used to improve Pap adherence. Because of this, we ask that you answer our questions in a sincere manner”.**

1. Explain how the focus group will proceed, including the various themes that will be discussed, how long the focus group will last, and that it will be **recorded**. Explain the focus group rules:
   1. Everybody can and should participate.
   2. Everybody should listen to and respect others’ opinions.
   3. Nobody can “win” or “lose”.
   4. There are no “good” or “bad” answers.
2. Explain that before the focus group begins, we will give each participant an incentive in the form of a gift card.
3. Clarify the confidentiality of the focus group, including informed consent, focus group recording, and the use of pseudonyms.

**HAVE THE STUDY PARTICIPANTS READ AND SIGN THE INFORMED CONSENT DOCUMENT. GIVE 10 MINUTES FOR THEM TO COMPLETE THE SHORT QUESTIONNAIRE.**

**Participant introduction –** each participant introduces themselves (10 min)

**CELL PHONE USE AND MOBILE TECHNOLOGY (5-10 MIN)**

**Introduction: We would like to begin by asking how you use your cellphones.**

1. What do you use your cell phone for?
2. Who uses your cell phone?
3. How many times in the past year have you had to change your cell phone number? Why?
4. How many of you send text messages? What for?
5. How many of you receive text messages? What kind?
6. What kinds of problems have you had with your cell phone? (I.e. running out of money, losing it, having to share it, etc.)

**GENERAL PERCEPTIONS REGARDING THE USE OF CELLPHONES AND TEXT MESSAGES IN HEALTH CARE (30 MIN)**

**Introduction: Many people have and use cellphones, and we would like to know if cellphones could be used to improve women’s health.**

1. How would you like health care personnel (midwives, doctors) to communicate with you?
2. What are your thoughts on communicating with them via text messaging? [Explore the use of reminder messages, real-time communication with health care personnel (uni/bi-directional), information on cervical cancer…]
3. Who would you like to receive text messages from? (doctors, nurses, midwives, no preference)
4. What do you think about us sending you text messages automatically from a computer directly to your cellphone? [Explore what characteristics that message should have]

**For example, we could automatically program educational messages to be sent to your phones that have recommendations for preventing cervical cancer.**

1. What do you think about receiving educational messages via text messaging? Would it be useful? Why or why not? What would you want the short text message to say? What other kinds of messages would you want to receive? [explore messaging related to nutrition, sexual health, alarm signs, motivation, physical activity, prevention, etc]
2. Frequency: How many messages would you want to receive per week? Is there a day in particular you prefer?
3. Are there any potential problems you could anticipate with receiving text messages? How would you feel if another person (ie family member or friend) accidentally read your text messages? [explore if there are confidentiality or privacy concerns]
4. What is your opinion regarding the idea of receiving automatic appointment reminders via text messaging? Would it be useful? Why or why not? What would you want the text message to say?
5. What is your opinion regarding the idea of receiving automatic Pap test reminders via text messaging? Would it be useful? Why or why not? What would you want the text message to say? How often would you want to receive them?
6. In total, how many messages per week would you want to receive? How would you want to be able to stop receiving messages?

**CONCLUSION (3 MIN)**

**Your answers will be very helpful for our study. Before we end, is there anything else you think could help us improve our study?**

**Thank you so much for your honest answers and time!**
