# Supplementary material for: Development of mobile technologies for the prevention of cervical cancer in Santiago, Chile study protocol: a randomized controlled trial
Source: BMC Cancer. 2017 Dec 13;17:847. doi: 10.1186/s12885-017-3870-8 (PMC5729241; doi:10.1186/s12885-017-3870-8)
Supplement: Additional file 1: — Focus group guides in English and Spanish. (ZIP 226 kb) [file 12885_2017_3870_MOESM1_ESM.zip › Supplemental Material 2-Focus groups guides- SpanishR2.docx]

PROYECTO MENSAJES PARA TU SALUD

**GUION PARA LOS GRUPOS FOCALES (GF) CON LOS PROFESIONALES**

**Objetivo GF:**

Conocer las barreras y facilitadores de una intervención basada en mensajería de texto para mantener la adherencia al PAP.

Describir el cuidad habitual.

INTRODUCCION (5 MIN)

**Pasos a seguir por el facilitador:**

1. Dar la bienvenida al grupo

2. Presentación del equipo organizador

3. Explicar el objetivo del grupo focal (GF), por qué han sido seleccionados e

invitados, por qué son importantes para el estudio. Ej.

*“El objetivo de la reunión es conocer lo que ustedes hacen habitualmente para mejorar la adherencia al Pap y conocer su opinión sobre que tipo de mensaje debiéramos enviar a sus usuarias. Por ello solicitamos a ustedes que sus respuestas sean sinceros”*.

4. Explicar la metodología de trabajo (discusión del tema, duración**, grabación**) y dar a conocer las reglas de la discusión: a. Todos pueden y deben participar. b. Todas las ideas y respuestas son válidas.

c. Cada persona debe escuchar y respetar la opinión de las otras.

d. Nadie gana ni pierde en el grupo.

e. No hay respuestas malas ni buenas

5. Aclarar la confidencialidad (consentimiento informado, **grabación**, uso de seudónimos).

**Presentación de cada uno de las participantes (10 min)**

(Nombre)

PREGUNTAS (30 MIN)

**Introducción: Queremos partir por preguntarles sobre el cuidado habitual de las mujeres que requieren un Pap.**

Qué tipos de actividades promocionales hace usted para incentivar a las mujeres a recibir un Pap?

Recibió entrenamiento extra para algo que se trata de la toma de Pap después de empezar a trabajar como matrona/matrón?

Cómo debiera ser una intervención basada en mensajería de texto para mejorar la adherencia a Pap (que deberían decir los mensajes, cuanto en seguidos debemos enviarlos, deberían las mujeres poder contestarlos, etc.)

Cuales son las posibles barreras y cómo pudiéramos mejorarlas?

Qué podría ser positivo sobre este tipo de intervención?

Hay algo más que quisieran decir?

CONCLUSION (3 MIN)

**Su información nos será de mucha ayuda para mejorar nuestro programa. Antes de que terminemos, ¿hay algo más que usted quiere compartir con nosotros?**

**Muchas gracias por ser honesto con nosotros, y por su tiempo!**

PROYECTO MENSAJES PARA TU SALUD

**GUION PARA LOS GRUPOS FOCALES (GF) CON LAS MUJERES**

**Objetivo GF:**

Conocer las percepciones de las mujeres sin Pap al día acerca del uso de celulares y mensajes de texto como manera de mejorar la adherencia al Pap.

INTRODUCCION (5 MIN)

**Pasos a seguir por el facilitador:**

1. Dar la bienvenida al grupo

2. Presentación del equipo organizador

3. Explicar el objetivo del grupo focal (GF), por qué han sido seleccionadas e

invitadas, por qué son importantes para el estudio. Ej.

***“El objetivo de la reunión es conocer lo que ustedes piensan sobre el uso de celulares y mensajes de texto para el cuidado de su salud. La opinión de cada una de ustedes es muy valiosa porque nos ayudara a entender ciertas cosas relacionadas al uso de celulares y como estas tecnologías pueden ser utilizados para mejorar su adherencia al Pap. Por ello solicitamos a ustedes que sus respuestas sean sinceras”*.**

4. Explicar la metodología de trabajo (discusión del tema, duración**, grabación**) y dar a conocer las reglas de la discusión:

a. Todas pueden y deben participar.

b. Todas las ideas y respuestas son válidas.

c. Cada persona debe escuchar y respetar la opinión de las otras.

d. Nadie gana ni pierde en el grupo.

e. No hay respuestas malas ni buenas

5. Explicar que antes de participar, entregamos un incentivo económico en forma de una tarjeta de regalo.

6. Aclarar la confidencialidad (consentimiento informado, **grabación**, uso de seudónimos).

**Leer el consentimiento informado y solicitar firma y dar 10 min Para contestar el cuestionario corto.**

**Presentación de cada una de las participantes (10 min)**

(Nombre)

USO DE CELULAR Y TECNOLOGIA MOVIL (5-10 MIN)

**Introducción: Queremos partir por preguntarles como usan su celular.**

**1. Para qué usa su celular?**

**2. Quién usa su celular?**

**3. Cuantas veces ha tenido que cambiar el número de celular en el último año? Por qué?**

**4. Cuantas de ustedes envían mensajes de texto? Para que?**

**5. Cuantas de ustedes reciben mensajes de texto? (sondeo general – *muy breve*) de que tipo?**

**6. Qué problemas han tenido del uso de su celular? (quedarse sin dinero, perderlo, compartirlo, etc.)**

PERCEPCIONES GENERALES SOBRE EL USO DE CELULARES Y MENSAJES DE TEXTO PARA LA SALUD (30 MIN)

**Introducción: Muchas personas tienen y usan celulares, y queremos saber si los celulares se pueden utilizar para el mejor cuidado de salud de las mujeres.**

1. ***Como les gustaría a ustedes que el personal de salud (matronas, médicos) se comuniquen con ustedes?***

2. ***Que opinan de comunicarse por mensajes de texto?***

***[Explorar uso de recordatorios, comunicación real con personal de salud [uni-bi-direccional], información sobre el cáncer cervicouterino,, ... ]***

***3. De quien les gustaría recibir mensajes de texto (explorar, médicos, enfermeras, matronas, no tienen ninguna preferencia)***

4. Y que tal si nosotros les enviamos automáticamente mensajes a través de una computadora directamente a su celular? .[explorar: que característica tendría que tener ese mensaje de texto]

**Por ejemplo, podemos programar automáticamente en enviarles mensajes educativos con recomendaciones sobre los cuidados que debe tener para prevenir el cáncer cérvico uterino sus celulares por mensaje de texto**

¿Qué opinan de la idea de recibir **mensajes educativos** a través del celular mediante mensajes de texto? Lo consideran útil? Si/No Por que? **Que te gustaría que dijera el mensaje corto?**

**Que otros tipos de mensajes les gustaría recibir? (explorar: nutricionales, salud sexual, signos de alarma, motivacionales, actividad física, preventivos, etc.)**

**Frecuencia: Cuantos mensajes a la semana? Existe algún día en particular preferido?**

Hay algún problema potencial que ustedes verían en recibir mensajes de texto? **¿**Como se sentiría si otra persona lee **(accidentalmente)** sus mensajes del celular (ejemplo. un familiar o un amigo)? *[****Explorar*** *si hay alguna preocupación por mantener la* ***confidencialidad*** *de los datos, a alguien le preocupa la* ***privacidad****]*

5. ¿Qué opinan de la idea de hacerles **recordar** automáticamente sus **citas** en el centro a través del celular con un mensaje de texto? Lo consideran útil? Si/No Por que?

**Que les gustaría que diga el mensaje corto?**

6. ¿Qué opinan de la idea de hacerles **recordar** automáticamente la **toma del Pap** a través del celular con un mensaje de texto? Lo consideran útil? Si/No Por que?

**Que te gustaría que diga el mensaje corto? Frecuencia?**

***En total:***

Cuantos mensajes a la semana les gustaría recibir?

**Como les gustaría dejar de recibir los mensajes de texto?**

CONCLUSION (3 MIN)

**Su información nos será de mucha ayuda para mejorar nuestro programa. Antes de que terminemos, ¿hay algo más que usted piense que pueda ayudarnos a mejorar el programa de celulares? Para usted o para otras personas como usted?**

**Muchas gracias por ser honesto con nosotros, y por su tiempo!**
